# Alzheimer’s disease phospholipase C-gamma-2 (PLCG2) protective variant is a functional hypermorph

**DOI:** 10.1186/s13195-019-0469-0

**Published:** 2019-02-02

**Authors:** Lorenza Magno, Christian B. Lessard, Marta Martins, Verena Lang, Pedro Cruz, Yasmine Asi, Matilda Katan, Jamie Bilsland, Tammaryn Lashley, Paramita Chakrabarty, Todd E. Golde, Paul J. Whiting

**Affiliations:** 1 UCL Alzheimer’s Research UK, Drug Discovery Institute, London, UK; 2 Department of Neuroscience, Center for Translational Research in Neurodegenerative Disease, and McKnight Brain Institute, College of Medicine, University of Florida, Gainesville, FL USA; 3 Research Department of Structural and Molecular Biology, University College London, London, UK; 4 Present address: Instituto de Medicina Molecular - João Lobo Antunes, Faculdade de Medicina de Lisboa, Lisbon, Portugal; 5 Department of Neurodegenerative Diseases, UCL Queen Square Institute of Neurology, University College London, London, UK; 6 Queen Square Brain Bank for Neurological Disorders, Department of Movement Disorders, UCL Queen Square Institute of Neurology, University College London, London, UK; 7 Dementia Research Institute, UCL, London, UK

**Keywords:** Neuroinflammation, Phospholipase C, Dementia, Immune response, Genetic variants

## Abstract

**Background:**

Recent Genome Wide Association Studies (GWAS) have identified novel rare coding variants in immune genes associated with late onset Alzheimer’s disease (LOAD). Amongst these, a polymorphism in phospholipase C-gamma 2 (PLCG2) P522R has been reported to be protective against LOAD.

PLC enzymes are key elements in signal transmission networks and are potentially druggable targets. PLCG2 is highly expressed in the hematopoietic system. Hypermorphic mutations in PLCG2 in humans have been reported to cause autoinflammation and immune disorders, suggesting a key role for this enzyme in the regulation of immune cell function.

**Methods:**

We assessed PLCG2 distribution in human and mouse brain tissue via immunohistochemistry and *in situ* hybridization. We transfected heterologous cell systems (COS7 and HEK293T cells) to determine the effect of the P522R AD-associated variant on enzymatic function using various orthogonal assays, including a radioactive assay, IP-One ELISA, and calcium assays.

**Results:**

PLCG2 expression is restricted primarily to microglia and granule cells of the dentate gyrus. *Plcg2* mRNA is maintained in plaque-associated microglia in the cerebral tissue of an AD mouse model. Functional analysis of the p.P522R variant demonstrated a small hypermorphic effect of the mutation on enzyme function.

**Conclusions:**

The PLCG2 P522R variant is protective against AD. We show that PLCG2 is expressed in brain microglia, and the p.P522R polymorphism weakly increases enzyme function. These data suggest that activation of PLCγ2 and not inhibition could be therapeutically beneficial in AD. PLCγ2 is therefore a potential target for modulating microglia function in AD, and a small molecule drug that weakly activates PLCγ2 may be one potential therapeutic approach.

**Electronic supplementary material:**

The online version of this article (10.1186/s13195-019-0469-0) contains supplementary material, which is available to authorized users.

## Background

Alzheimer’s disease is the most common neurodegenerative disorder and the leading cause of dementia. Late onset AD is genetically complex, and known susceptibility loci only explain a proportion of disease heritability [1]. Large-scale genetic studies have led to the identification of several new susceptibility genes associated with LOAD. These genetic data, together with an analysis of biological pathways, implicate processes related to immune response in the etiology of LOAD and point to the immune system as a prime target for therapeutic approaches [2–4]. Amongst the newly discovered polymorphisms, rare variants in microglial-related genes including triggering receptor expression on myeloid cell-2 (TREM2), ABI family member 3 (ABI3), and phospholipase C-gamma-2 (PLCG2) have been described [5, 6]. Notably, the PLCG2 missense variant Pro522Arg (*P* = 5.38 × 10^−10^, OR = 0.68) was associated with decreased risk of LOAD [6].

PLCG2 belongs to the family of phospholipase C-gamma and encodes an enzyme (PLCγ2) that cleaves the membrane phospholipid PIP2 (1-phosphatidyl-1D-myo-inositol 4,5-bisphosphate) to secondary messengers IP3 (myo-inositol 1,4,5-trisphosphate) and DAG (diacyl-glycerol) which further propagate a wide range of downstream signals. PLCγ2 shares high structural and mechanistic overlap with the other member of the PLCγ family, PLCγ1 [7]. Although both enzymes are important for regulation of specific responses of specialized cells of the immune system, they show different cell-type expression and are relevant in very different medical conditions [7]. PLCG1 is ubiquitously distributed, and mutations are associated with some forms of cancers, such as cutaneous T cell lymphoma [8, 9], but also neuropsychiatric disorders [10]. PLCγ2 is predominantly expressed in the bone marrow and lymphoid organs (Human Protein Atlas available from www.proteinatlas.org [11]), and PLCG2 variants cause inherited immune disorders designated as PLAID (PLCG2-associated antibody deficiency and immune dysregulation [12] and APLAID (autoinflammatory PLAID [13]).

In peripheral immune cells, PLCγ2 has been implicated in signaling pathways downstream of the B cell receptor, and it is thought to modulate the functions of macrophages, platelets, mast cells, neutrophils, and NK cells through the Fc receptor [14].

PLCγ2 belongs to the same interaction network as TREM2 [6] and may be directly involved in its signaling pathway [15, 16]. TREM2 is a transmembrane receptor expressed on the membrane of myeloid cells and an important susceptibility gene for AD [5]. In osteoclasts, activation downstream Trem2 initiates a cascade of events, including phosphorylation and activation of PLCγ2, amongst other proteins [17]. In microglia, these pathways promote several cellular responses including survival, proliferation, phagocytosis, enhanced secretion of cytokines, and chemokines [18] and are potentially involved in neurodegenerative processes.

Genomic studies can accelerate the pathways to novel therapeutics by suggesting promising translational targets and informing the type of approach to be undertaken. The identification of naturally occurring genetic variants with protective effects against diseases represents a valuable potential resource for drug development [19, 20].

The P522R polymorphism lies within the regulatory domain of PLCγ2, but its effect on enzyme function is unknown. Given that the variant is protective against LOAD, understanding its effect is fundamental in determining whether a small molecule inhibitor or activator/stabilizer would be beneficial as a therapeutic.

PLCγ2 spatial expression in resident brain immune cells and in AD is unexplored. Therefore, we first characterized its distribution in the brain. We found that *Plcg2* mRNA mainly co-localizes with microglia markers in healthy brain tissue, as well as in microglia near amyloid plaques in an amyloid precursor protein (APP) mouse model of AD. Additionally, functional characterization of the AD protective variant PLCγ2 p.P522R revealed a small increase in activity compared to wild type (WT) enzyme. PLCγ2 is therefore a potential target for modulating microglia function in AD, and a small molecule drug that activates PLCγ2 may be one potential therapeutic approach.

## Methods

### Animals

WT mice were maintained on a C57BL6 background at the Wolfson Institute for Biomedical Research in accordance with UK legislation (ASPA 1986).

TgCRND8 mice were maintained in-house by breeding APP transgenic males (carrying WT RD gene [21] with C57B6/C3H F1 females (Envigo). These mice have florid AD-type Aβ plaque pathology in their forebrains, starting around 3 months of age. Animal procedures were approved by the University of Florida Institutional Animal Care and Use Committee. All animals were house grouped, under standard laboratory conditions (12:12-h light/dark cycle, lights on at 0600 h) with a room temperature of 21 °C, and water and food available ad libitum.

### Mouse tissue processing, immunohistochemistry (IHC), and *in situ* hybridization (ISH)

IHC was carried out as previously described [22]. Primary antibodies used were the following: rabbit anti-PLCγ2 (1:50, H160, Santa Cruz Biotechnologies sc-9015), rabbit anti-PLCγ2 (custom produced and purified by Pacific Immunology Corp, Ramona, CA, using the peptide sequence “INSLYDVSRMYV”), rabbit anti-Iba-1 (ionized calcium binding adaptor molecule 1, 1:500, Q08578, Alpha Laboratories), and rabbit anti-NeuN (Neuronal Nuclei, 1:500, ABN78, Millipore). Secondary antibodies (Alexa, Invitrogen) were used at a final dilution of 1:1000.

Adult mice were perfusion-fixed with 4% paraformaldehyde (PFA), and the brains were dissected out and post-fixed overnight in 4% PFA. Samples were cryoprotected by overnight immersion in 20% sucrose, embedded in optimal cutting temperature compound (Tissue Tek, Raymond Lamb Ltd., Medical Supplies, Eastbourne, UK) and frozen on dry ice. Fifteen μm-thick cryosections were collected onto Superfrost slides and ISH (RNAScope Multiplex Fluorescent v2 323110, ACD Bio-Techne) was carried out according to manufacturer’s instructions with the following catalog probes: Mm-Plcg2 474781; Mm-Plcg1-C2 483531-C2; Mm-Olig2-C2 447091-C2; Mm-Pecam1-C3 316721-C3; Mm-Slc1a3-C2 430781-C2; Mm-Trem2-C2 404111-C2 (ACD, Bio-Techne). For some experiments, ISH was followed by IHC with anti-Iba1.

All sections were counterstained with Hoechst 33258 dye (Sigma, 1000-fold dilution), and the slides were mounted with Dako Fluorescence Mounting Medium (DAKO).

Confocal images (*z* stack height on average 10 μm, 1 μm spacing) were taken on a Zeiss LSM 880 confocal microscope (Carl Zeiss AG) and processed for contrast and brightness enhancement with Photoshop (CS5, Adobe). A final composite was generated in Adobe Illustrator (CS5, Adobe).

RNASCope reaction was quantified using CellProfiler Software [23] on confocal images (2/3 fields per animal per staining) taken with 40× objective. Dots per cell (nucleus) were counted, and the numbers of cells co-expressing mRNA (> 1 dot per cell) for *Plcg2* and other markers were calculated. Due to the use of nuclear staining to identify cell boundaries, this method does not allow to assess for transcripts quantification in processes.

### Human tissue processing, IHC, and ISH

Post-mortem human frontal cortex formalin-fixed, paraffin-embedded, 5-μm-thick sections were supplied by Queen Square Brain Bank for Neurological Disorders, UCL-Institute of Neurology (London, UK). The tissue is stored for research under a license from the Human Tissue Authority. Sections were deparaffinized in xylene and rehydrated in graded ethanol dilutions (100%, 90%, and 70%). Endogenous non-specific peroxidase activity was quenched by 0.03% H_2_O_2_ (Sigma-Aldrich) in methanol (1:100 dilution) for 5 min, followed by a 10-min antigen retrieval treatment in a pressure cooker. After blocking with 10% non-fat milk solution for 1 h at RT, slides were incubated with the primary antibody (rabbit anti-PLCγ2 H160, Santa Cruz Biotechnologies sc-9015) overnight at 4 °C (1:50). Sections were incubated with biotinylated anti-rabbit IgG (1:200, Sigma-Aldrich) for 30 min at RT. After three 5-min washes in PBS, sections were incubated with avidin-biotin complex horseradish peroxidase (ABC) reagent (Thermo Fisher Scientific) for 30 min. The reaction was developed with diaminobenzidine (DAB) activated by H_2_O_2_. Sections were counterstained with Mayer’s hematoxylin (RAL Diagnostics). Sections were dehydrated though increasing concentrations of ethanol (70%, 90%, and 100%) and cleared in xylene series. Finally, slides were mounted with DPX (Sigma-Aldrich) and cover slipped.

ISH on human tissue was carried out on frozen sections. After thawing, slides were fixed with 4% PFA for 25 min at RT, prior to hybridization with the following catalog probes: Hs-PLCG2 506781, HS-PPIB positive control 313901, and DapB negative control 310043. The protocol was carried out with a Multiplex Fluorescent v2 kit (323110, ACD Bio-Techne) following manufacturer instruction, with an added final incubation step for 30 s with Trueblack Lipofuscin Autofluorescence Quencher (Insight Biotechnology Ltd) to quench endogenous fluorescence.

### Cell culture and transfections

Plasmids for the expression of full-length human pEGFPC1-PLCG2 constructs in mammalian cells have been described previously [24]. PLCG2 is from Origene ([25]). EGFR (epidermal growth factor receptor) construct is a gift from Axel Ullrich (Addgene plasmid # 65225). QuikChange PCR mutagenesis (Stratagene) was used to introduce the P522R point mutation in the PLCG2 common variant. All mutants were fully sequenced to verify the fidelity of the PCR reaction.

COS7 cells were maintained at 37 °C in a humidified atmosphere of 95% air and 5% CO_2_ in Dulbecco’s modified Eagle’s medium (DMEM, Invitrogen) supplemented with 10% (*v*/*v*) fetal bovine serum (Invitrogen) and 2.5 mM glutamine. Prior to transfection, cells were seeded into 6-well plates at a density of 2.5 × 10^5^ cells/well and grown for 16 h in 2 ml/well of the same medium. For transfection, 1 μg of PLCG2 plasmid DNA was mixed with 1 μl of PlusReagent™ and 7 μl of Lipofectamine™ (Invitrogen) and added to the cells in 0.8 ml of DMEM without serum. The cells were incubated for 3.5 h at 37 °C, 5% CO_2_ before the transfection mixture was removed and replaced with DMEM-containing serum.

### Analysis of inositol phosphate formation in intact COS7 cells

Inositol phosphate formation was assessed as described in [26]. This is an established, standardized assay that has been, amongst other applications, successfully used to assess previously identified disease-linked variants of PLCG2 [12, 13, 26].

Briefly, 24 h after transfection, the cells were washed twice with inositol-free DMEM without serum and incubated for 24 h in 1.5 ml of the same medium supplemented with 0.25% fatty acid free bovine serum albumin (Sigma) and 1.5 μCi/ml myo-^2-3^[H] inositol (MP Biomedicals). After a further 24 h, the cells were incubated in 1.2 ml of inositol-free DMEM without serum containing 20 mM LiCl with or without stimulation with 100 ng/ml EGF (Epidermal Growth Factor, Calbiochem). The cells were lysed by addition of 1.2 ml of 4.5% perchloric acid. After incubating the samples on ice for 30 min, they were centrifuged for 20 min at 3700 × *g*. Supernatants and pellets were separated. The supernatants were neutralized by addition of 3 ml of 0.5 M potassium hydroxide/9 mM sodium tetraborate and centrifuged for a further 20 min at 3700 × *g*. Supernatants were loaded onto AG1-X8 200–400 columns (Bio-Rad) that had been converted to the formate form by addition of 2 M ammonium formate/0.1 M formic acid and equilibrated with water. The columns were washed three times with 5 ml of 60 mM ammonium formate/5 mM sodium tetraborate, and inositol phosphates were eluted with 5 ml of 1.2 M ammonium formate/0.1 M formic acid. Five milligrams of Ultima-Flo scintillation fluid (PerkinElmer Life Sciences) was added to the eluates and the radioactivity quantified by liquid scintillation counting. The values represent total inositol phosphates. The pellets from the first centrifugation were resuspended in 100 μl of water, and 375 μl of chloroform/methanol/HCl (200:100:15) was added. The samples were vortexed, and an additional 125 μl of chloroform and 125 μl of 0.1 M HCl were added.

After further vortexing, the samples were centrifuged at 700 × g for 10 min. Ten microliters of the lower phase were placed in a scintillation vial with 3 ml of Ultima-Flo scintillation fluid and the radioactivity quantified by liquid scintillation counting. The obtained values correspond to radioactivity in inositol lipids. PLC activity is expressed as the total inositol phosphates formed relative to the amount of [^3^H]myo-inositol in the phospholipid pool. Because the differences in steady-state labeling of inositol lipids are small (within 20%), this normalized PLC activity corresponds closely to PLC values expressed as total inositol phosphates.

### Western blotting

PVDF membranes were blocked in TBS 0.5% casein 1 h at RT. Antibodies (anti-GFP, 11814460001, Roche; rabbit anti-PLCγ2 custom produced, rabbit anti-EGFR D38B1, 4267, Cell Signaling Technologies) were diluted in TBS with 0.2% Tween-20 (TBS-T) and incubated 1 h at RT. The membranes were washed with TBS-T and Western blots analyzed with Odyssey infrared imaging system (LI-COR Inc., NE, USA).

### ELISA D-myo-inositol 1-phosphate

D-myo-inositol 1-phosphate (IP1) was quantified by ELISA IP-One (CISBIO US, Inc., USA) according to the manufacturer’s instructions. Briefly, HEK293T co-transfected with EGFR and PLCG2 were stimulated with human recombinant EGF (Thermo Fisher, Carlsbad, USA) 150 ng/ml for 1 h. Cell lysates were loaded in ELISA plate for IP1 quantification.

### Calcium flux assay

This assay has been successfully used to assess the function of transiently transfected PLCG1 and 2 [13, 27]. Intracellular calcium (Ca2+) changes were measured with FURA-2-AM (Invitrogen, USA) based on [28]. Transiently transfected HEK293T cells attached to poly-l-lysine-treated plate were washed with HBSS (HBSS-Hepes buffer; 120 mM NaCl, 5.3 mM KCl, 0.8 mM MgSO4, 1.8 mM CaCl2, 10 mM glucose, 20 mM Hepes, pH 7.4) and loaded with Fura 2-AM (Sigma) for 20 min at RT in the dark. Cells were washed with HBSS and incubated with HBSS for 30 min at RT. The intracellular Ca2+ changes were measured by alternating the fluorescence excitation wavelengths 340 and 380 nm and emitted fluorescence at 510 nm. Data acquisition was typically at 5-s intervals, and data were presented as emitted fluorescence ratio 340/380.

### Statistical analysis

Statistical analysis was performed using Prism 6 version 6.05 for Windows (GraphPad Software, La Jolla, CA, USA). Data were analyzed using an appropriate statistical test followed by post hoc one-way ANOVA with Tukey’s multiple comparison test and two-way ANOVA with Bonferroni multiple comparisons. Details of the statistical test used are included in the figure legends.

## Results

### PLCG2 is expressed in microglia cells throughout the brain

**Fig. 1:**
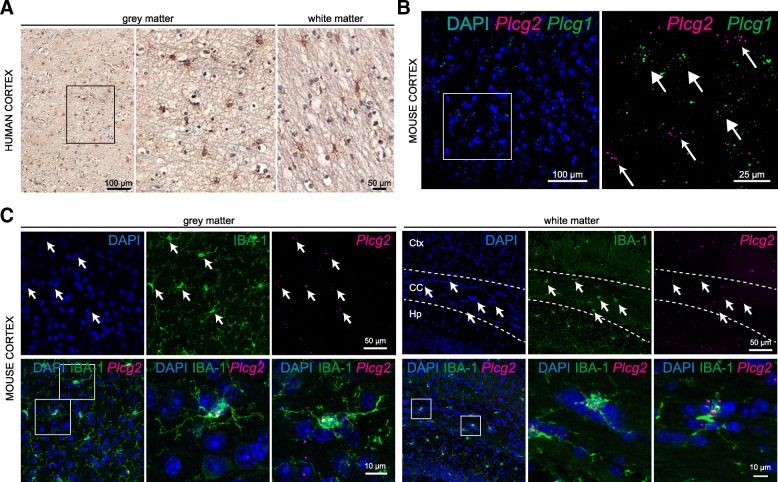
PLCγ2 expression in the human and mouse cortical microglia. **a** IHC for PLCγ2 on the gray and white matter of the human prefrontal cortex. **b** Multiplex RNAScope for *Plcg2* and *Plcg1* on the adult mouse cortex. Small and big arrows point to RNASCope reaction for *Plcg2* and *Plcg1*, respectively. **c** RNAScope for *Plcg2* followed by IHC for IBA-1 on the gray and white matter of the adult mouse cortex. Arrows point to co-expression of the two markers. *Ctx* cortex, *CC* corpus callosum, *Hp* hippocampus

Recent transcriptomic datasets suggest prominent *Plcg2* expression in microglia of the mouse cortex [29–31]; however, spatial mapping of the protein and mRNA in brain tissue has not been described. To better understand the involvement of this protein in AD, we assessed PLCγ2 distribution in human cortical tissue. We detected PLCγ2 immunoreactivity in putative microglia of both white and gray matter, in the human frontal cortex (Fig. 1a). Due to technical difficulties with the antibodies used in this study (in one case, the antibody is no longer commercially available; in other cases, antibodies gave unspecific or no signal upon IHC on fixed human post-mortem brain or mouse tissue), we turned to *in situ* hybridization (ISH, RNAScope, [32]). RNAScope on human cortex with a probe specific for PLCG2 confirmed the sparse distribution pattern observed with immunolabeling (Additional file 1a). We further investigated the expression of *Plcg2* across the mammalian brain. Since PLCγ1 seems to be expressed ubiquitously in mammalian tissues, while PLCγ2 is thought to be restricted to the hematopoietic and immune system [7], we sought to determine the distribution of these two PLCs in the brain. We found that *Plcg1* and *Plcg2* mRNAs show complementary expression patterns, with very little degree of overlap in the adult mouse brain (Fig. 1b). Labeling for *Plcg1* was detected in several brain cells, including large nuclei of putative neurons of the cortex (Fig. 1b). Conversely, *Plcg2* reaction product was found in sparse cells, in cortical as well as subcortical regions, including the olfactory bulb, neocortex, hippocampus CA1, and substantia nigra (Additional file 1b).

In line with the single-cell RNA-seq profiling databases of the mouse cortex (see above), we confirmed a high degree of co-localization between *Plcg2* mRNA and the microglia-specific marker IBA-1 in cortical gray and white matter of the adult mouse brain. In these regions, all IBA1-immunoreactive microglia cell bodies contained transcripts for *Plcg2* (100%, Fig. 1c). We further validated high degree of co-expression between the two markers in subcortical brain regions, including the thalamus and the cerebellum (data not shown).

To determine whether other brain cell types express *Plcg2*, we quantified co-localization between *Plcg2* and other glial/endothelial cell markers. We found only occasional co-expression with markers for astrocytes (*Glast* GLutamate ASpartate Transporter/Slc1a3 Solute Carrier family 1 member 3, 5.8 ± 3.8%, Additional file 2a), endothelial cells (*Pecam1*, platelet and endothelial cell adhesion molecule 1, 9.6 ± 1.44%, Additional file 2b), and oligodendrocyte-lineage cells (*Olig2*, oligodendrocyte transcription factor 2, 1.6 ± 2.2% Additional file 2c).

In addition to a generally scattered labeling in most brain areas, we found intense *Plcg2* RNAScope reaction product in the granule cell layer of the hippocampal dentate gyrus, where the *Plcg2* signal co-localizes with the neuronal marker NeuN (Additional file 2d). Rare co-labeling with NeuN was also detected in other subcortical regions (not shown). In summary, *Plcg2* transcripts are primarily restricted to microglia cells across the mouse brain, with the exception of the pronounced expression seen in granule cells in the dentate gyrus.

### PLCG2 in microglia in Alzheimer’s disease

*PLCG2* mRNA upregulation has been reported in cortical tissue of LOAD patients (FC, 1.35; *p* = 0.0028, [33]) and in transgenic mice carrying mutations associated with early AD (APP KM670/671 NL, PNSEN1 M146V) or overexpressing the human tau-4R/2N isoform (P301L) [34, 35]. However, normalization to microglia-specific genes (e.g., Abi3) and data from single-cell profiling of brain immune cells [6, 36, 37], suggest that apparent PLCG2 transcript upregulation in bulk tissue is mostly related to microgliosis occurring as a consequence of neurodegeneration.

**Fig. 2:**
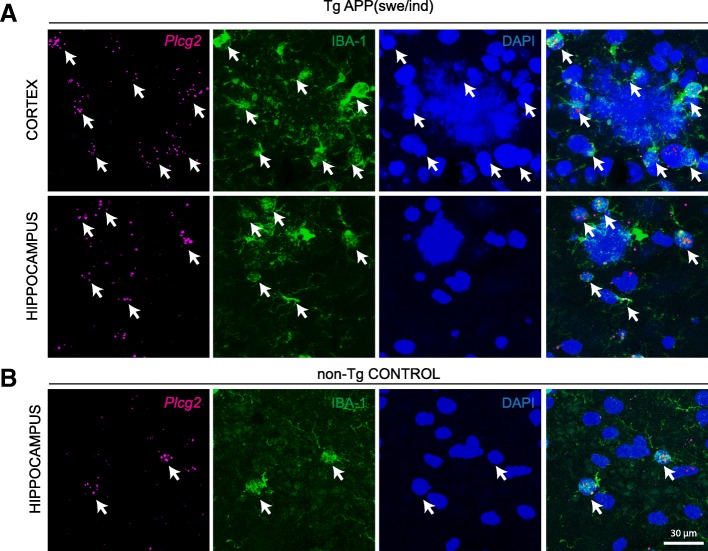
*Plcg2* mRNA in microglia at amyloid plaques in a mouse model of AD. **a** RNAScope for *Plcg2* in adult mouse neocortex and hippocampus of TgAPPswe/ind transgenic mice. Note expression of *Plcg2* in IBA-1-labeled microglia surrounding the plaques (DAPI). Arrows point to co-labeling of IBA-1 and *Plcg2.* **b** RNAScope for *Plcg2* in adult mouse hippocampus of a non-transgenic control mouse. Arrows point to co-labeling of IBA-1 and *Plcg2*

Recent findings point to microglia heterogeneity in neurodegeneration [36, 38]. To determine whether heterogeneity in Plcg2 expression might be observed in microglia in AD, we carried out ISH for *Plcg2* on the brain of a transgenic mouse model overexpressing the human APP with the Swedish (KM670/671NL) and Indiana (V717F) mutations (TgAPPswe/ind [21]). *Plcg2* appeared to be equally expressed in microglia surrounding the plaques in the cortical regions of 6 months old transgenic mice (Fig. 2a) as compared to localization in microglia in control non-transgenic littermates (Fig. 2b).

**Fig. 3:**
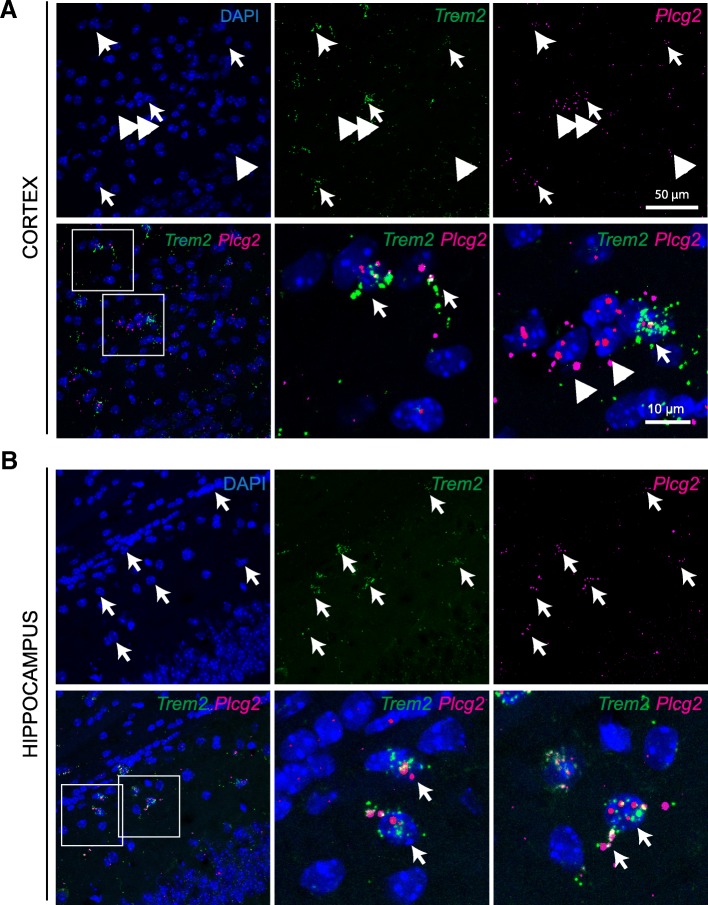
*Plcg2* and Trem2 transcripts co-localize in the mouse brain. **a** Multiplex RNAScope for *Plcg2* and *Trem2* in the adult mouse cortex*.* Arrows point to co-expression, arrowheads indicate single-labeled cells. **b** Multiplex RNAScope for *Plcg2* and *Trem2* in the adult mouse hippocampal CA1*.* Arrows point to co-expression, arrowheads indicate single-labeled cells. Note co-localization in processes of putative microglia

Next, we assessed whether in the brain Plcg2 co-localizes with the GWAS AD risk gene Trem2, as suggested by protein-protein interaction network models [6]. Indeed, we detected co-expression of *Plcg2* and *Trem2* mRNAs in the cell soma and processes of putative cortical and hippocampal microglia (Fig. 3).

### AD associated PLCG2 p.P522R variant shows a weak hypermorphic activity

Germline deletion and point mutations in PLCG2 cause complex immune disorders and autoinflammatory disease in humans [12, 13]. Similarly, two hypermorphic Plcg2 mutations in mice (Ali5, Abnormal limb 5, D993G; Ali14, Abnormal limb 14, Y495C) lead to severe autoinflammation and antibody deficiencies [26, 39, 40]. Stimulation of the PLC enzymes increases the hydrolysis of PIP2, producing IP3 (further converted to other inositol phosphates) and DAG. Binding of IP3 on its receptor induces intracellular Ca2+ release from the Ca2+ stored in endoplasmic reticulum. The effects of the above mutations on enzyme function have been determined by quantifying the production of inositol phosphates or the release of intracellular Ca2+ following stimulation with epidermal growth factor (EGF) in heterologous cell systems *in vitro* [7].

**Fig. 4:**
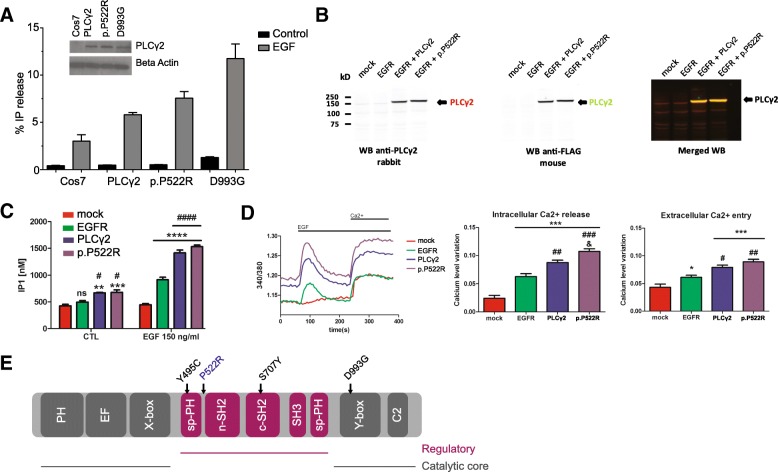
PLCγ2 protective variant p.P552R shows a slightly hypermorphic activity by increasing intracellular calcium release. **a** PLC activity of the p.P522R variant under basal and stimulated conditions. Measurement of % IP release in non-transfected COS7 cells (COS7), COS7 cells transfected with pEGFPC1-PLCG2 constructs (common variant, PLCG2 WT; AD associated, rare variant PLCG2 P522R; and PLCG2 D993G (Ali5) mutation. Western blotting was used to confirm equal expression (anti-GFP, inset). SD is represented by error bars. The data are representative for 3 independent experiments. **b** HEK293T were transfected with mock, EGFR, EGFR and PLCG2 WT, or EGFR and PLCG2 P522R plasmids. **c** Quantification of IP1 by ELISA IP-One. Transfected HEK293T cells were stimulated with EGF 150 ng/ml and analyzed for IP1 quantification. ELISA reading were averaged of ± standard error (*n* = 3, *p < 0.05, **p < 0.01, ***p < 0.001, ****p < 0.0001, #*p* < 0.05, ####*p* < 0.0001, two-way ANOVA, Bonferroni multiple comparisons, *compared to control, #compared to EGFR). **d** Transfected HEK293T cells loaded with Fura-2AM were stimulated with human EGF 150 ng/ml. Intracellular calcium level was recorded by calculating the 340/380 nm excitation ratio at each 5 s. The net intracellular Ca2+ releases value was obtained by subtracting the average of 340/380 excitation ratio values between 0 and 50 s from the maximum 340/380 excitation ratio value between 60 and 200 s, after the EGF stimulation. The net extracellular Ca2+ entry value was obtained by subtracting the average 340/380 excitation ratio values between 285 and 380 s from the average of 340/380 excitation ratio values between 215 and 235 s after adding the extracellular calcium. Data in **d** are shown as averages of ± standard error (*p < 0.05, **p < 0.01, ***p < 0.001, #*p* < 0.05, ##*p* < 0.01, ###*p* < 0.001, & *p* < 0.05, one-way ANOVA, Tukey’s multiple comparison test, *compared to mock, #compared to EGFR, & compared to PLCγ2. The “*n*” is corresponding to an average of 45 reading from 3 independent transfection). **e** Linear representation of the domains of PLCG2 with locations of the point mutations (Y495C, Ali14; P522R, AD protective variant; S707Y, APLAID; D993G, Ali5)

To investigate the effect of the AD protective variant P522R on PLCγ2 enzyme function, we firstly measured production of ^3^[H] inositol phosphates in a radioactive assay as described [26] upon transient transfection of COS7 cells with pTriEx4-PLCG2 (common, “wild type” variant; PLCG2 522P) construct, the AD associated (P522R) construct, and with the D993G mutation (Ali5 [39]) construct. PLC activity after stimulation with EGF showed a 1.24 ± 0.06 fold increase (mean value ± SD over 3 independent experiments, normalized to PLCγ2 WT activity) upon transfection with the P522R construct over the WT. For comparison, the inflammation-related Ali5 p.D993G mutation exhibited a larger (2.27 ± 0.43 fold, mean value ± SD) increase in enzyme function upon EGF stimulation (Fig. 4a).

Inositol phosphate IP1 accumulates over time and can be measured by ELISA. As a second functional endpoint, we therefore evaluated the enzyme activity of PLCγ2 p.P522R by measuring the cellular level of IP1 after activation of transfected epidermal growth factor receptor (EGFR) into HEK293T cells. We confirmed expression of the WT, the p.P522R variant, and EGFR (Fig. 4b, Additional file 3). Stimulation of mock transfected cells with EGF failed to increase IP1 level (Fig. 4c). The expression of EGFR or EGFR + PLCγ2 significantly increased the amount of IP1 confirming an activation of the receptor and PLCγ2. p.P522R enzyme activity was higher than PLCγ2, as measured by IP1 production (Fig. 4c).

As a third endpoint of PLCγ2 function, we measured real time changes in intracellular Ca2+. No Ca2+ changes were seen in mock HEK293T cells stimulated with EGF (Fig. 4d), confirming our previous ELISA IP-One results, and as previously observed [13]. Expression of EGFR induced significant intracellular Ca2+ changes after addition of EGF, as measured by increase of the 340/380 nm excitation ratio (Fig. 4d, left panel). Quantification of the intracellular Ca2+ release showed a small but clear increase for p.P522R variant as compared to WT PLCγ2 (Fig. 4d, middle panel). Depletion of the intracellular Ca2+ stores activates a Ca2+ flux across the plasma membrane, referred to as store-operated Ca2+ entry or capacitative Ca2+ entry [41, 42]. This Ca2+ flux was observed by exposing the cell to extracellular Ca2+ (Fig. 4d, right panel). Ca2+ level monitoring show no significant differences in the extracellular Ca2+ entry between cells expressing EGFR + PLCγ2 and EGFR + p.P522R (Fig. 4d, right panel).

## Discussion

Although single-cell transcriptomics of the human and mouse cortex suggest enrichment of *PLCG2* transcripts in the microglia population, no spatial analysis has been described for human and mouse tissue so far. In this report, we characterized *PLCG2* spatial expression in the human and mouse brain.

One technical limitation of this study is that we could not confirm mRNA and protein co-expression in the same cells. However, in human cortical tissue, we were able to show PLCγ2 immunoreactivity in sparsely located cells with microglia-like morphology, and *PLCG2* transcripts were detected in similarly sparsely distributed cells, reminiscent of microglia. Together with the data of *Plcg2* expression in mouse tissue, our results strongly point to co-expression of protein and mRNA, at least in microglia in the mammalian cortex.

In a modest, but significant number of cells in the mouse cortex *Plcg2* co-localized with a marker of endothelial cells. According to a transcriptomic analysis of cells in the vasculature, Plcg2 seems to be expressed in certain subtypes of endothelial cells, although at lower level than compared to microglia (http://betsholtzlab.org/VascularSingleCells/database.html [43]).

Another interesting finding is the detection of *Plcg2* in the granule cell layer of the dentate gyrus. The role of Plcg2 in these neurons is unknown. By analogy with Plcg1 function in neurons, Plcg2 in granule cells may be relevant for synaptic transmission and plasticity via induction of hippocampal long-term potentiation LTP [10, 44]. Given the involvement of the dentate gyrus in pattern separation—distinction of closely related memories—Plcg2 expression might be important for mnemonic functions. Interestingly, to date, no deficits in learning and memory have been described in Plcg2 −/− mice, or in humans bearing the PLCG2 hypermorphic variants.

We showed that microglia are the main cell brain population expressing Plcg2. The function of PLCγ2 in the immune system has been studied in relation to autoinflammatory and immune deficiency syndromes associated with PLCG2 mutations in humans and mice [12, 13, 39]. In the brain, microglia activation of Plcg2 might regulate intracellular calcium release, as shown for B cells [12, 45], and contribute to the modulation of specific immune responses such as phagocytosis, migration, proliferation, and survival. One receptor acting upstream of Plcg2 and evoking such responses in innate immune cells might be Trem2 [17, 46]. Indeed, human induced pluripotent stem cell microglia-like cells harboring mutations in TREM2 show distinct defects in phagocytosis [47]. We found apparent co-localization of *Plcg2* and *Trem2* transcripts in microglia processes, which suggests a possible requirement of local translation for quick responses to environmental changes, and a mechanism to detect neural tissue damage. Mutations in TREM2 have been associated with increased risk of AD [5, 18]. Moreover, increased levels of Trem2 have been detected in disease-associated microglia compared to microglia in a homeostatic state (37). We found Plcg2 expression in microglia surrounding amyloid plaques in a mouse model of AD as well as in “homeostatic” microglia. This pathway, and in particular PLCγ2 activity, could be critical for microglia function in neurodegenerative conditions. Other potential signaling links of PLCγ2 variants with upstream (ITGAM or FC receptors [14]) or downstream (e.g., the NRLP3 inflammasome [48]) components in microglia will be also addressed by our further studies.

Our functional analyses of the p.P522R variant reveal a small hypermorphic effect on enzyme activity. The molecular mechanism by which this occurs is currently unknown. Other mutations in PLCG2 that lead to immune dysfunction are known to be activating [7]. These mutations have been proposed to affect enzyme function by disruption of the autoinhibitory interface, destabilization of the regulatory domain that blocks enzyme inactivation, or alteration of interaction with the membrane. The protective variant P522R is certainly located within a region of the enzyme that has a regulatory function (Fig. 4d, [7]), which is consistent with this polymorphism modulating enzyme activation.

## Conclusion

In summary, we show that PLCG2 is expressed in human and mouse brain microglia and that its expression is maintained in microglia in close proximity to plaques in the cerebral tissue of an APP mouse model. The rare-coding AD protective variant shows a small hypermorphic activity upon stimulation in various orthogonal cell-based assays. Further experiments will need to address the functional consequences of the protective variant on immune cell phenotype. However, these findings allow us to speculate that weak lifelong activation of PLCγ2 might confer protection against developing AD, and provide evidence that a limited activation of this enzyme may have a beneficial therapeutic effect. In comparison to previous studies which are aligned to the concept that suppressing microglial function may be beneficial in the setting of AD [49], the data described here, together with studies on TREM2 and other AD-associated variants, suggest that a different directionality may be therapeutically useful [6, 46, 50].

## Additional files

Additional file 1: Plcg2 *in situ* hybridization on brain tissue. A) RNAScope for *PLCG2*, positive (PPIB) and negative (DapB) controls in human cortex. B) RNAScope for *Plcg2* in adult mouse brain (OB olfactory bulb, CA1 hippocampal area CA1, DG dentate gyrus, SNr substantia nigra pars reticulata). (PDF 2671 kb)

Additional file 2: Characterization of Plcg2 cell-type co-expression. A) Multiplex RNAScope for *Plcg2* and *Glast* followed by IHC for IBA-1 on adult mouse cortex*.* Arrowheads indicate single-labeled cells. B) Multiplex RNAScope for *Plcg2* and *Pecam1* on adult mouse cortex. Arrows point to co-expression of the two markers, arrowheads point to single-labeled cells. C) Multiplex RNAScope for *Plcg2* and *Olig2* on adult mouse cortex*.* Arrows point to co-expression of the two markers, arrowheads point to single-labeled cells. D) RNAScope for *Plcg2* followed by IHC for NeuN on dentate gyrus granule cell layer*.* (PDF 6277 kb)

Additional file 3: Assessment of EGFR and PLCG2 expression levels in HEK293T cells transfected with EGFR (Western blotting). (PDF 87 kb)

## Abbreviations

ABI3: ABI family member 3
Ali14: Abnormal limb 14
Ali5: Abnormal limb 5
APLAID: Autoinflammatory PLAID
APP: Amyloid precursor protein
DAG: Diacyl-glycerol
EGF: Epidermal growth factor
GLAST: Glutamate aspartate transporter
GWAS: Genome Wide Association Studies
IBA1: Ionized calcium binding adaptor molecule 1
IHC: Immunohistochemistry
IP1: D-Myo-inositol 1-phosphate
IP3: Myo-inositol 1,4,5-trisphosphate
ISH: In situ hybridization
LOAD: Late onset Alzheimer’s disease
NeuN: Neuronal nuclei
Olig2: Oligodendrocyte transcription factor 2
Pecam-1: Platelet and endothelial cell adhesion molecule
PFA: Paraformaldehyde
PIP2: 1-Phosphatidyl-1D-myo-inositol 4,5-bisphosphate
PLAID: PLCG2-associated antibody deficiency and immune dysregulation
PLCG2: Phospholipase C-gamma 2
Slc1a3: Solute carrier family 1 member 3
TgAPPswe/ind: Transgenic mouse line with APP with the Swedish (KM670/671NL) and Indiana (V717F) mutations
TREM2: Triggering receptor expression on myeloid cell-2
WT: Wild type
