# Supplementary figures and images for: Alzheimer’s disease phospholipase C-gamma-2 (PLCG2) protective variant is a functional hypermorph
Source: Alzheimers Res Ther. 2019 Feb 2;11:16. doi: 10.1186/s13195-019-0469-0 (PMC6359863; doi:10.1186/s13195-019-0469-0)

A

human cortex

*PPIB* + DAPI*DABP* + DAPI*Plcg2* + DAPI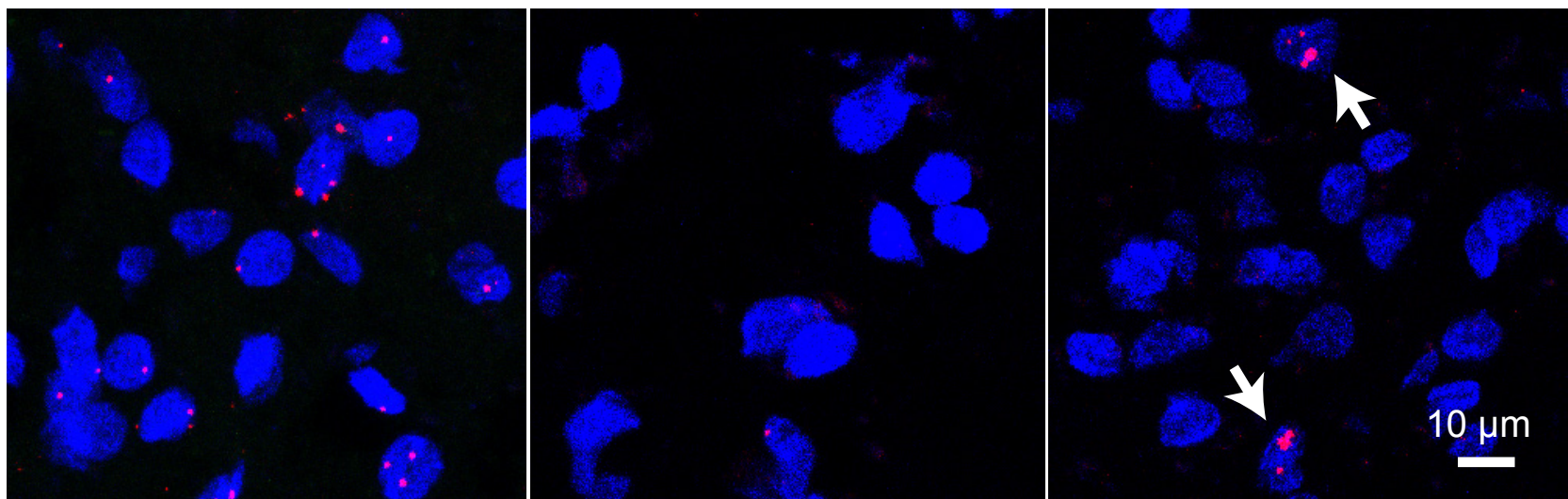

mouse brain

B

*Plcg2* + DAPI

OB

CA1

DG

SNr

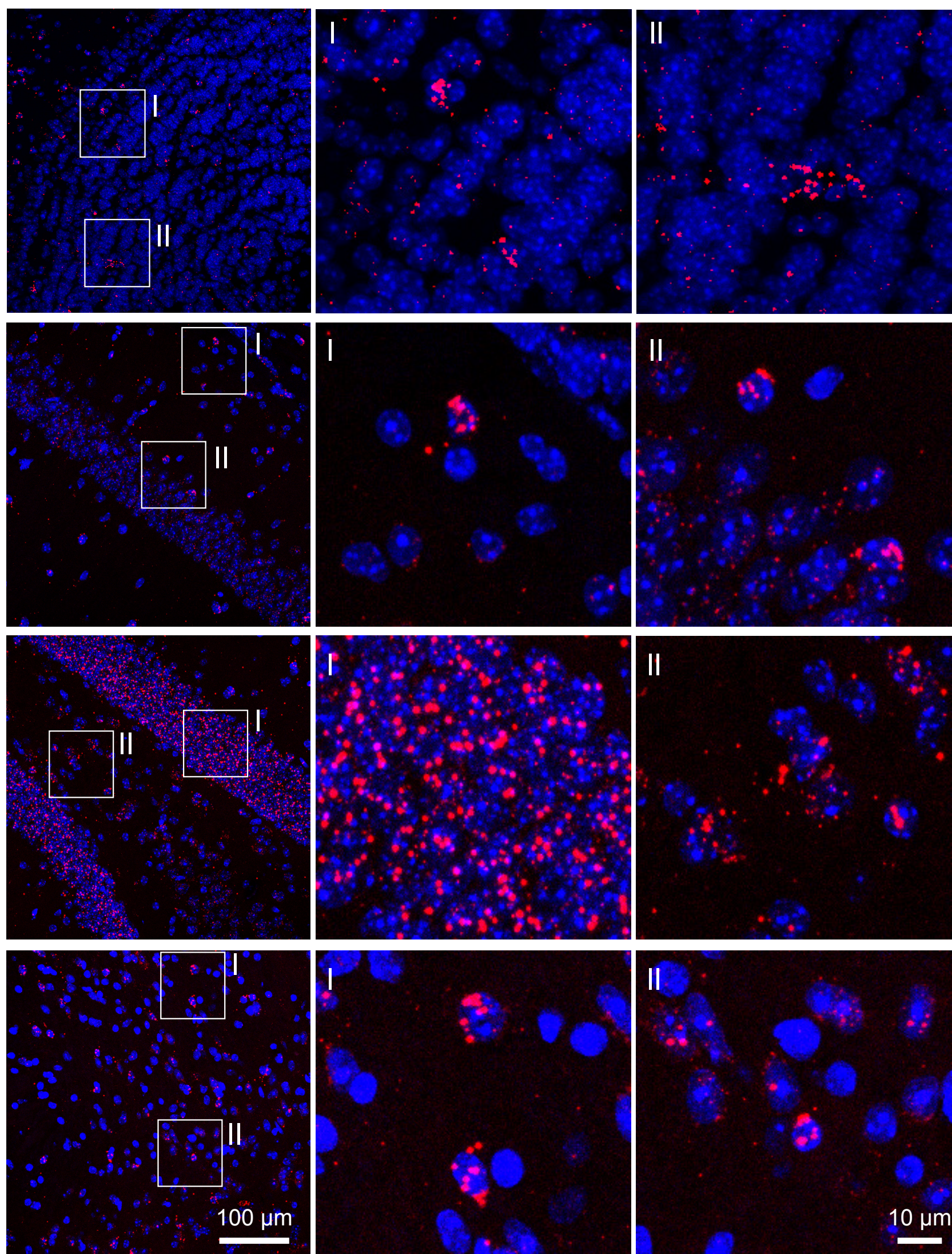

Supplement: Supplementary file 1 — Plcg2 in situ hybridization on brain tissue. A) RNAScope for PLCG2, positive (PPIB) and negative (DapB) controls in human cortex. B) RNAScope for Plcg2 in adult mouse brain (OB olfactory bulb, CA1 hippocampal area CA1, DG dentate gyrus, SNr substantia nigra pars reticulata). (PDF 2671 kb) [file 13195_2019_469_MOESM1_ESM.pdf]

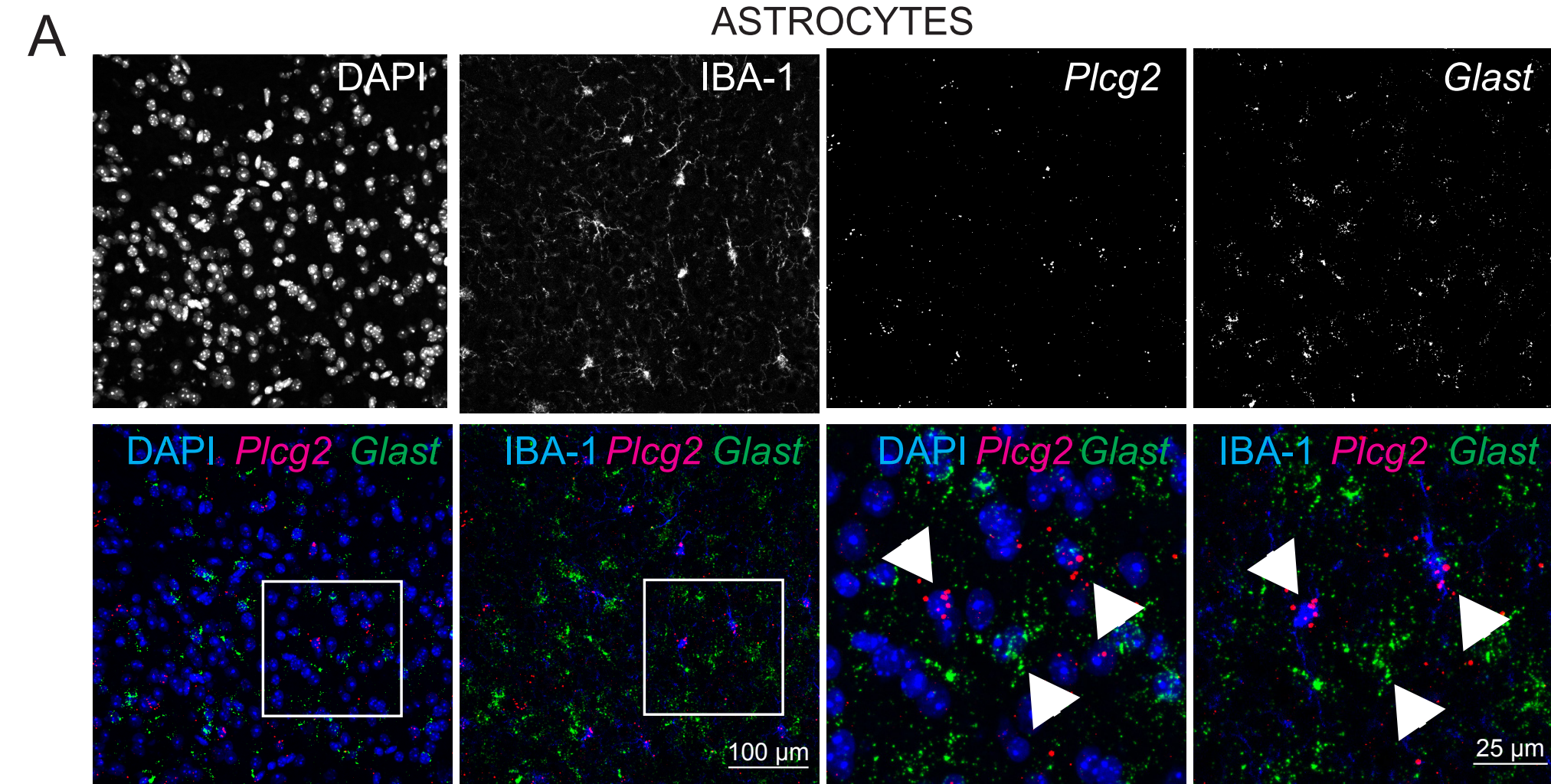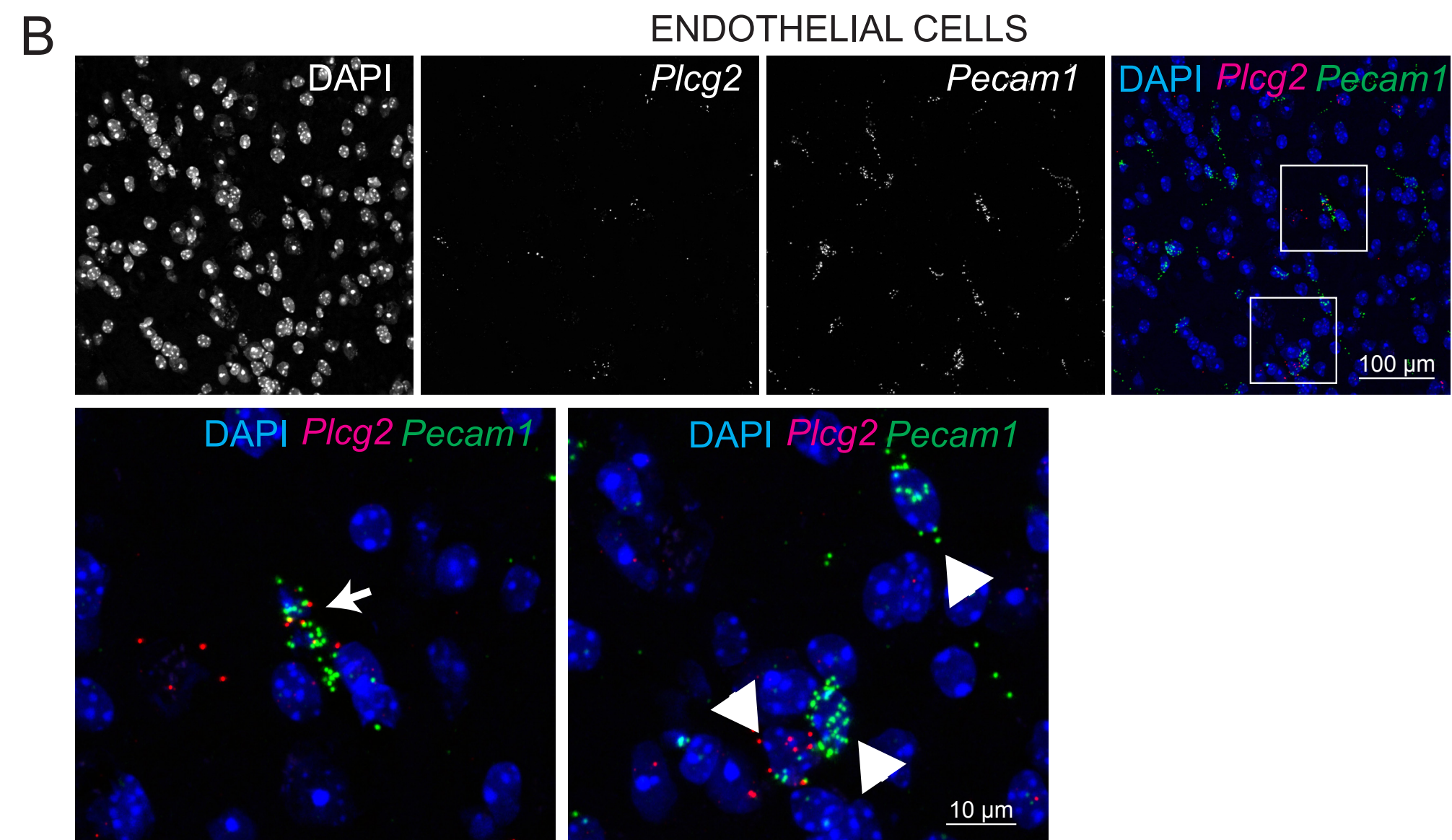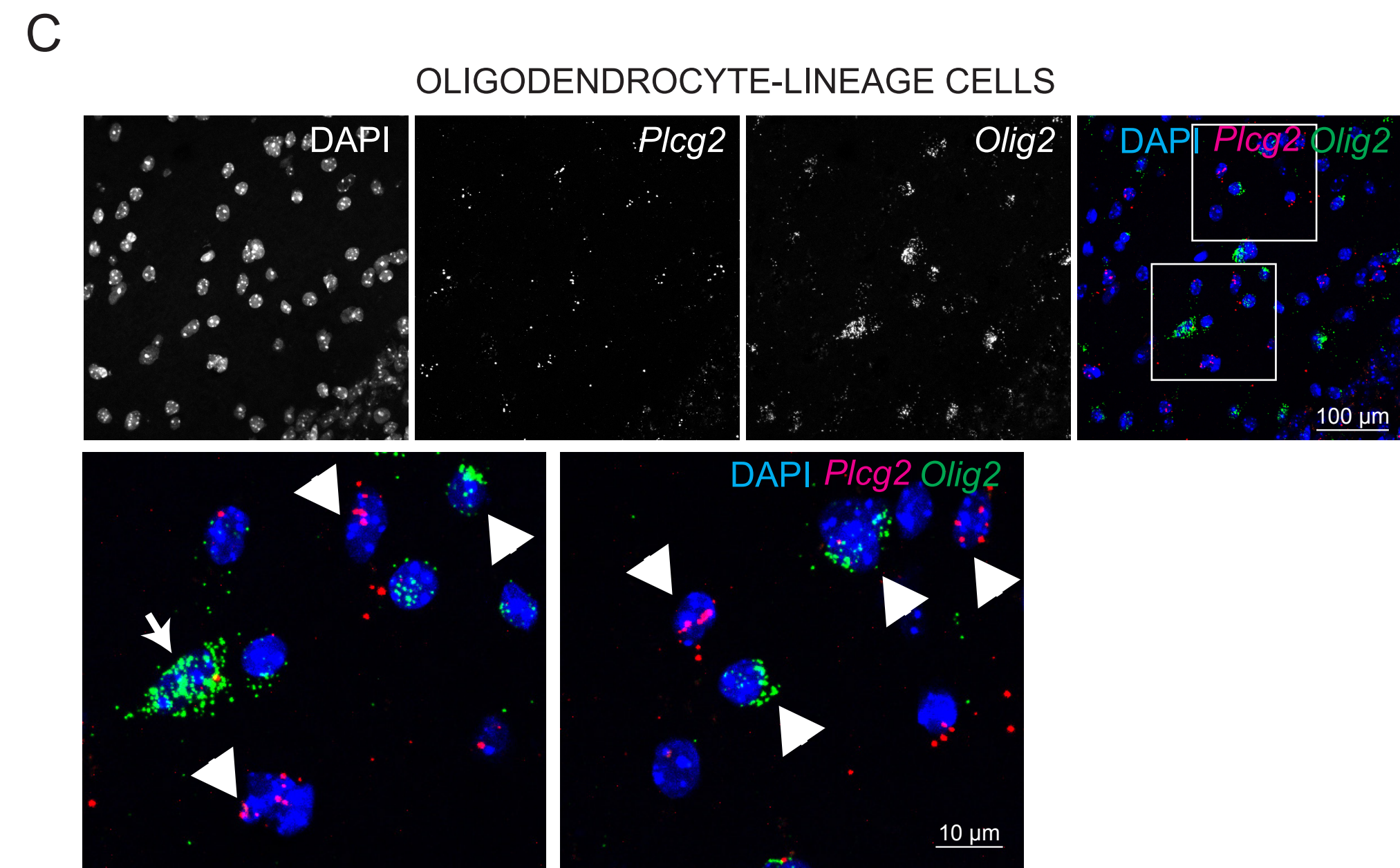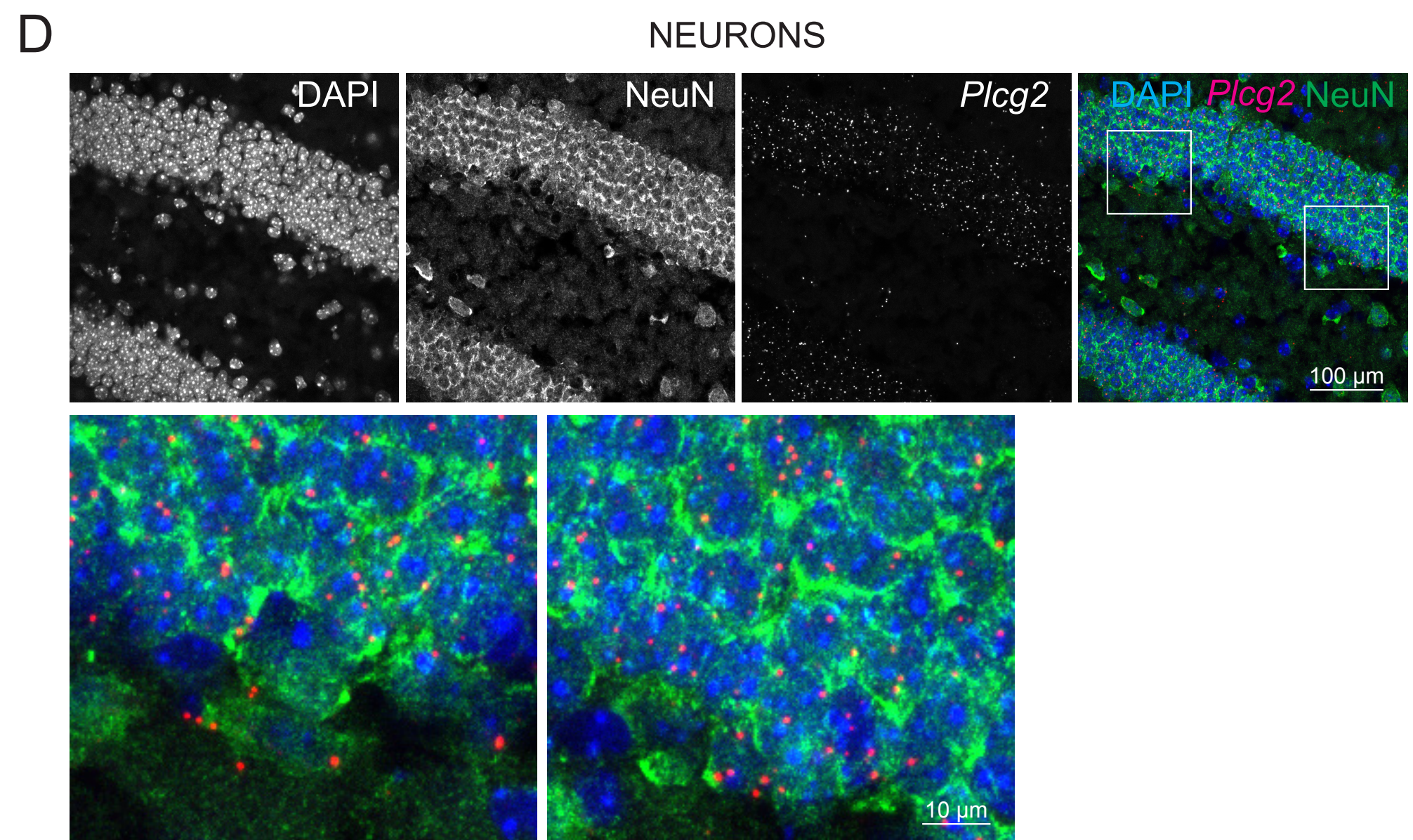

Supplement: Supplementary file 2 — Characterization of Plcg2 cell-type co-expression. A) Multiplex RNAScope for Plcg2 and Glast followed by IHC for IBA-1 on adult mouse cortex. Arrowheads indicate single-labeled cells. B) Multiplex RNAScope for Plcg2 and Pecam1 on adult mouse cortex. Arrows point to co-expression of the two markers, arrowheads point to single-labeled cells. C) Multiplex RNAScope for Plcg2 and Olig2 on adult mouse cortex. Arrows point to co-expression of the two markers, arrowheads point to single-labeled cells. D) RNAScope for Plcg2 followed by IHC for NeuN on dentate gyrus granule cell layer. (PDF 6277 kb) [file 13195_2019_469_MOESM2_ESM.pdf]

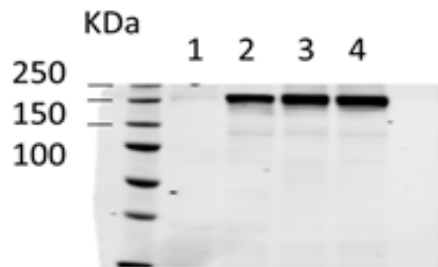

**EGFR**

- 1 - mock**
- 2 - EGFR**
- 3 - EGFR + PLCγ2**
- 4 - EGFR + p.P522R**

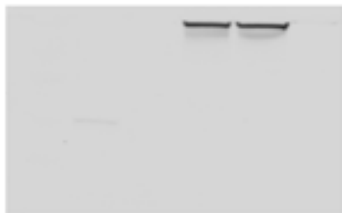

**PLCγ2**

Supplement: Supplementary file 3 — Assessment of EGFR and PLCG2 expression levels in HEK293T cells transfected with EGFR (Western blotting). (PDF 87 kb) [file 13195_2019_469_MOESM3_ESM.pdf]
